# Fertility, Pregnancy and Lactation Considerations for Women with CF in the CFTR Modulator Era

**DOI:** 10.3390/jpm11050418

**Published:** 2021-05-15

**Authors:** Raksha Jain, Jennifer L. Taylor-Cousar

**Affiliations:** 1 Internal Medicine, Pulmonary and Critical Care, University of Texas Southwestern Medical Center, Dallas, TX 75390, USA; Raksha.Jain@UTSouthwestern.edu; 2 National Jewish Health, Internal Medicine and Pediatrics, Pulmonary, Denver, CO 80206, USA

**Keywords:** pregnancy, contraception, fertility, lactation, CFTR modulator

## Abstract

Cystic fibrosis (CF) is an autosomal recessive genetic disorder impacting approximately 80,000 people of all races and ethnicities world-wide. CF is caused by mutations in the cystic fibrosis transmembrane conductance regulator (CFTR) gene which encodes a protein of the same name. Protein dysfunction results in abnormal chloride and bicarbonate transport in mucus membranes, including those in the respiratory, gastrointestinal and reproductive tracts. Abnormal anion transport causes viscous secretions at the site of involvement. The majority of people with CF succumb to respiratory failure following recurrent cycles of infection and inflammation in the airways. Historically, providers treated the signs and symptoms of CF, but since 2012, have been able to impact the basic defect for the subset of people with CF who have mutations that respond to the new class of drugs, CFTR protein modulators. With the improved health and longevity afforded by CFTR modulators, more women are interested in parenthood and are becoming pregnant. Furthermore, this class of drugs likely increases fertility in women with CF. However, the safety of CFTR modulators in pregnancy and lactation is only beginning to be established. We summarize available data on the impact of CFTR modulators on fertility, pregnancy and lactation in women with CF.

## 1. Introduction

Cystic Fibrosis (CF) is a rare genetic disease caused by mutations in a gene called the CF Transmembrane conductance Regulator (CFTR), which codes for a chloride and bicarbonate transport protein located on the surface of epithelial cells in organs throughout the body. Lack of chloride transport is associated with lack of sodium and water transport, resulting in dehydrated surfaces and thickened secretions, such as purulent mucus in the lungs and obstructed pancreatic ducts. Sequela of these thickened secretions is a multi-system disorder that usually includes pancreatic insufficiency and bronchiectasis and progressive respiratory failure among many other complications. In countries in which CF is included in newborn screening panels, the majority of people with CF are diagnosed in the first months of life, although missed early diagnosis may result in diagnosis in adulthood [1,2,3]. Over 2000 variants have been described in the CFTR gene, the overwhelming majority of which occur in less than 1% of those affected by CF [4]. Up to 90% of people with CF carry at least one copy of the most common mutation, Phe508del (F508del) [3,5]. In 2012, the first oral therapy to treat CFTR at the protein level was approved called ivacaftor (IVA) for a subset of people with certain mutations [6]. This therapy increased chloride transport dramatically and transformed the health of eligible people with CF. Over the next several years, 3 other combination CFTR protein modulators, called lumacaftor/ivacaftor (LUM/IVA), tezacaftor/ivacaftor (TEZ/IVA) and, most recently, elexacaftor/tezacaftor/ivacaftor (ETI) became available for the majority of people with CF with responsive CFTR mutations based on data demonstrating increased lung function, improved quality of life and decreased pulmonary exacerbations among other benefits [7,8,9,10,11]. With these and many other therapies, the life expectancy and quality of life for people with CF is dramatically improving with a median life expectancy now in the upper 40s; further increases are expected in the next decade [3].

## 2. Fertility

The majority of women with CF are able to conceive and carry out a pregnancy to term, however, infertility may occur secondary to CF. Unfortunately, this phenomenon has not been systematically studied in CF and the exact prevalence of infertility in women with CF is unknown. Some data show a reported prevalence of infertility or subfertility of approximately 20–35%, which is potentially 10–20% higher than that in the general population [12,13,14]. In a multicenter French study, median time to conception in women with CF was reported as 12 months [15]. Unlike men with CF who are almost always azoospermic, due to congenital bilateral absence of the vas deferens [16], the structural anatomy of the reproductive system of women with CF is similar to that of women without CF. The infertility in women with CF is hypothesized to be due to a number of potential factors which may or may not be related to CF as described below.

### 2.1. Possible Causes of Infertility in Women with CF

While the exact etiology of infertility in women with CF is unknown, there are several possible causes that may be associated with their underlying disease process. Some women with CF have irregular menstrual cycles, though the exact reason and prevalence relative to that of the general population is unknown. Historically, menarcheal delay, abnormal ovulation and even amenorrhea were thought to be associated with low body weight and malnutrition leading to hypothalamic suppression [17]. As nutrition has improved as a whole in people with CF with appropriate use of pancreatic enzymes and nutritional supplements, and particularly since the introduction of CFTR modulators, malnutrition occurs less frequently and delayed puberty is less common in adolescents with CF [3,18,19]. Reduced ovarian reserve has also been described in CF, but it is unclear if there is a direct association between this occurrence and CFTR expression in the ovaries [20,21].

A more clear and direct cause of infertility in women with CF relates to the abundance of CFTR expression on the epithelial cells of the cervix [22,23,24]. Epithelial cells in the reproductive tract are impacted by CFTR gene mutations as they are in the lungs and other organs. With limited chloride and other ion transport resulting in thickened secretions [22,25,26], women with CF may have thick, dehydrated, cervical mucus resulting in impaired ability for sperm to penetrate the cervical os. Furthermore, defective CFTR alters bicarbonate secretion, resulting in a pH-imbalanced environment, which can result in failure of sperm capacitation and potential prevention of fertilization of the egg in some women with CF [22,26,27].

### 2.2. Unexpected Pregnancies and Improved Fertility with Modulators

Reports indicate that women with CF lack thorough knowledge regarding their fertility [28,29,30]. Studies of women with CF indicate confusion about how CF affects fertility and pregnancy, with women perceiving that they are infertile or have low fertility [29]. This lack of understanding of female fertility in women with CF is further supported in Polish and Australian cohorts of women, who also indicated that they believed their fertility to be reduced [28,31]. In association with this lack of understanding and education surrounding infertility, sexually active young women with CF report that they are less likely to use contraception than women without CF [30]. The decreased contraceptive use may be an indication that women with CF underestimate their ability to conceive.

Based on the presence of CFTR channels in the uterus and cervix, it was hypothesized that fertility for women with CF will improve with the availability of CFTR modulators and, specifically, highly effective CFTR modulators (IVA and ETI). Case reports of unplanned pregnancies suggest that fertility is improved with use of IVA [32,33,34,35]. Additionally, there is a recent case series from the United States supporting this theory [34]. Two CF care centers reported that 14 women with CF conceived after initiation of ETI, 7 of which were unplanned and 4 of which occurred in women who had been previously deemed infertile following clinical evaluation. The exact effect of CFTR modulators on fertility is not yet known, but they are thought to decrease viscosity and increase pH in cervical mucous secretions, promoting a more fertile environment. While exact numbers are not yet available, reports of unexpected pregnancies have occurred on ETI [34,35].

### 2.3. Fertility Case Example

**Figure 1:**
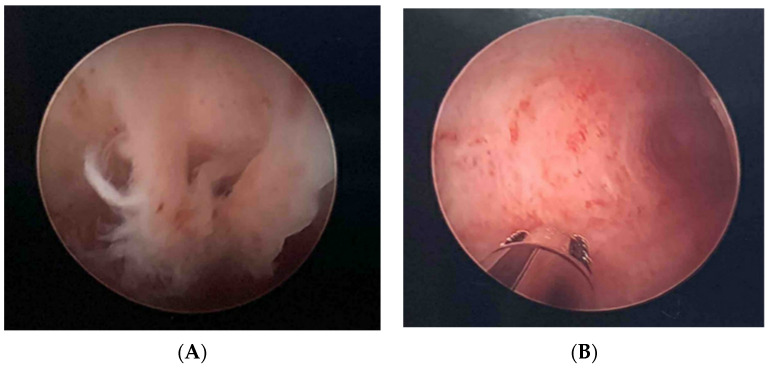
(**A**) Hysteroscopic image of the uterus of a woman with CF prior to initiation of CFTR modulator. (**B**) Image of the uterus in the same woman 3 months after initiation of IVA.

A 31-year-old woman with a percent predicted forced expiratory volume in one second (ppFEV1) of 82, body mass index (BMI) of 22.1 kg/m^2^, history of chronic cough, frequent bronchitis and chronic sinusitis presented to her Obstetrician (OB) after she and her husband had been trying to get pregnant for nearly 2 years without success. She underwent a number of tests including uterine examination via hysteroscopy. Her OB discovered what were described as “bands” of mucus in the woman’s uterus and cervix, and the woman was consequently deemed infertile (Figure 1A). Based on these findings, she was referred to a CF clinic and found to have sweat tests of 63 and 64 mmol/L and two pathogenic CFTR variants (c.1021_1022dupTC (p.F342fs*28) and c.328G>C (p.D110H). Based on these results, she was diagnosed with CF, started on standard airway clearance therapies for her chronic cough and on the CFTR modulator, IVA based on her mutation eligibility. Within a few days, she felt her general health to be dramatically improved, and noticed a clearance of sinus and respiratory secretions as well as a vaginal purge of mucus. On follow up with her OB several months later, her OB was impressed by the near resolution of the bands of thick mucus (Figure 1B) and has advised the couple to pursue a natural conception at this time.

## 3. Pregnancy

### 3.1. History of Pregnancy in Women with CF

**Figure 2:**
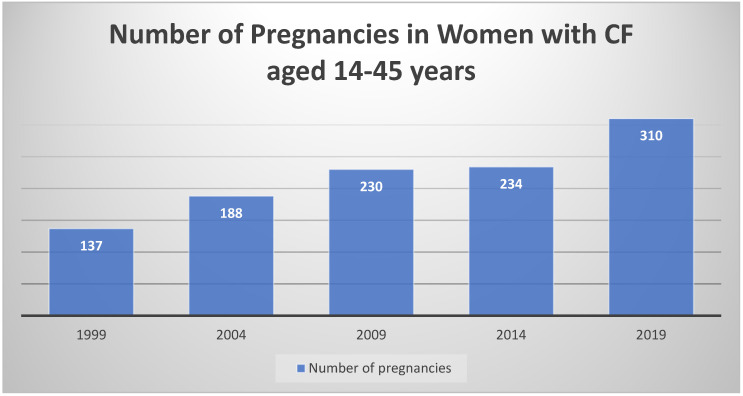
The number of pregnancies reported in the U.S. CFFPR [3] The number of pregnancies in women with CF has been increasing over time.

Thirty years after the first pathological description of cystic fibrosis occurred, the first pregnancy in a woman with CF was reported. Unfortunately, the woman died of respiratory failure less than 2 months after the birth of her 34-week-old infant; it was felt that her pregnancy substantially accelerated her disease progression, and consequently, caution was advised for women with CF considering pregnancy [36]. More modern data continues to suggest that for women with moderate to severe CF lung disease, there is increased risk of premature delivery of neonates who also have a higher incidence of complications [37,38,39,40]. Babies born to mothers with CF also have a higher rate of congenital anomalies compared to the incidence in women without CF [38]. Another risk factor for maternal and infant complications, diabetes, occurs in approximately 30% of adults with CF [3,41]. Importantly for women with CF, investigators have shown that in spite of some decline in health during pregnancy, with our current management, women with CF do not experience accelerated disease progression [40,42]. The impact of CFTR modulators on the health of the pregnant mother and her infant is not yet known. On the other hand, in the era of CFTR modulators, the median predicted survival for people with CF is 46 years in the U.S., with prospects for even greater longevity with the widespread use of highly effective CFTR modulator therapy [3,43]. In this setting of improved health and optimism about the future, young women with CF are expressing an increased desire to bear children [30], and pregnancy rates in women with CF are increasing [3] (Figure 2).

### 3.2. Pregnancy Case Example

A 37-year-old woman with CF genotype F508del/F508del and moderate CF lung disease (baseline ppFEV1 61%) presented to clinic with her husband of 10 years to discuss initiation of ETI. Previous genetic testing for her husband revealed no CFTR mutations. She stated that she had never gotten pregnant despite not using birth control at any point in their marriage. The likelihood of increased fertility (described above) was discussed as part of standard pre-ETI counseling.

Five months following transition from LUM/IVA to ETI, she called the clinic to state that she was 5 weeks pregnant. A telehealth visit was scheduled (COVID pandemic) to review what is known about the use of CFTR modulators during pregnancy, and the potential benefits and risks to her and her developing fetus of continuing or stopping modulator therapy. Because her health had improved dramatically on ETI, and based on concerns about potential deterioration, she elected to continue ETI throughout her pregnancy. On her in-person follow-up visit at week 9 of her pregnancy, her ppFEV1 was 75% and her weight had increased from 56.5 kg to 60.8 kg. Her pregnancy was complicated by one abnormal fetal ultrasound suggesting possible small fetal pericardial effusion (later determined by cardiology to be of no clinical significance). She experienced no pulmonary or sinonasal exacerbations during her pregnancy. Her labor lasted approximately 13 h, and ultimately resulted in a Cesarean section because of shoulder dystocia. Her healthy baby girl was born at 39 weeks.

Again, taking into consideration her own health and the limited amount of data regarding lactating women and the use of CFTR modulators, she elected to continue use of ETI while lactating. At the woman’s post-pregnancy follow up CF clinic visit, her ppFEV1 was 80%. She reported that her baby is growing well, and has had no jaundice. The infant’s newborn screen was normal. The baby has not yet had a cataract exam, and her pediatrician elected not to check liver function tests in the setting of the normal growth and good health of the infant.

### 3.3. Data from Animal Reproductive Models Following CFTR Modulator Administration

Pregnant women are almost always excluded from participation in Phase III trials because of known or unknown risks to the fetus of study drug administration. In 2015, the federal drug administration (FDA) changed the requirements for labeling new therapeutics with regards to risks of the drug for the developing fetus or lactating infant [44]. Under the new rule, sponsors must describe; (1) whether there are adequate and well-controlled studies in pregnant women to say if there is a drug-associated risk of major birth defects or miscarriage; and (2) studies in animal reproductive models including the amount of drug administered compared to the maximum recommended human dose (MRHD), and fetal impact of such dosing. Each of the CFTR modulators, IVA, LUM, TEZ and ELX, have been tested in animal reproductive models [45,46,47,48]. (See Table 1). Such testing showed that even at toxic human doses, there was no adverse impact of the individual CFTR modulators on fetal chromosomes, organogenesis or survival. However, of note, when juvenile rates (aged 7–35 days) were directly administered IVA, the development of neonatal cataracts was observed at all doses. Thus, use of IVA or products that contain IVA (LUM/IVA, TEZ/IVA and ETI) in children requires baseline and yearly ophthalmologic examination. In the U.S., IVA is approved for infants 4 months of age and older and LUM/IVA is approved for children ≥2 years [45]. Post-approval monitoring for cataract development in children exposed to IVA or IVA combination therapy is on-going.

### 3.4. Data in Pregnant Women Exposed to CFTR Modulators during Pregnancy

While data from animal models is reassuring, there are no adequately controlled studies of use of CFTR modulators in pregnancy. However, all 4 modulators are expected to cross the placenta and therefore expose the developing fetus to drug [45,46,47,48]. In fact, in a mother who continued LUM/IVA throughout pregnancy, Trimble and colleagues measured LUM and IVA in maternal and infant plasma and cord blood [49]. Concentrations of CFTR modulators in cord blood exceeded (LUM) or were equivalent (IVA) to those that were observed in maternal plasma. Thus, in counseling women with CF who are considering pregnancy or who are pregnant, as with every drug that pregnant women with CF use to maintain their health, the health benefits to the mother of continuing the drug must be weighed against both the risk to her health if she discontinues the therapy and the known and unknown risks to the developing fetus if she continues the therapy. While there is adequate experience during pregnancy with many of the drugs used in the treatment of CF [50,51], to date, all information regarding use of CFTR modulators in pregnancy has been generated from case reports, case series and two surveys of CF providers [32,33,35,49,52,53,54,55,56].

The first reports of women with CF whose infants were exposed to a CFTR modulator during pregnancy occurred following approval of IVA [32,52,53]. All of the women were heterozygous for the IVA-responsive G551D mutation and had mild CF lung disease. They delivered healthy infants. In 2017, Vekaria and colleagues described the case of a woman with severe lung disease (ppFEV1 < 50) who conceived and delivered healthy infants in two separate pregnancies [54]. Subsequent case reports of women with mild to moderate disease included women who delivered healthy babies exposed to LUM/IVA during pregnancy [33,49,55]. More recently, Nash et al. described the results of a survey to CF care providers regarding 64 pregnancies in 61 women with CF who were intentionally or inadvertently exposed to IVA (*n* = 31), LUM/IVA (n-26) or TEZ/IVA (*n* = 7) for all or part of their pregnancies [56]. The first trimester miscarriage rate was 4.7%, lower than expected in the general U.S. population [57]. Two of the providers surveyed reported maternal complications that, in their opinions, were related to CFTR modulator use (one instance of a pulmonary exacerbation and one instance of acute myelocytic leukemia, both in women on LUM/IVA). Importantly, and consistent with previous reports of clinical decline following discontinuation of CFTR modulators [58,59], 9 women experienced health deterioration following IVA or LUM/IVA discontinuation, leading their providers to restart therapy during pregnancy. No providers reported infant complications related to CFTR modulator use, although very few infants underwent formal ophthalmologic exams.

Although the data from this survey was reassuring because it was collected prior to the approval of ETI, no information on ETI use was collected. As approximately 90% of women with CF are eligible for this therapy based on its effectiveness for those with at least one copy of F508del, information regarding the use of ETI during pregnancy and lactation is needed. The authors modified the survey utilized to collect data from CF clinicians regarding women who used the previously approved modulators, to collect data on 47 women who used ETI during some portion of their pregnancy and/or lactation [35]. Interestingly, both the range of baseline lung function and age range were wider in women who were exposed to ETI during pregnancy (29–122 and 21–41, respectively) versus that of those exposed to IVA, LUM/IVA and TEZ/IVA (48–106 and 21–34, respectively). As with the use of IVA, LUM/IVA and TEZ/IVA, the first trimester miscarriage rate for women on ETI was lower (8.9%) than that reported for women in the general U.S. population [56,57]. One maternal complication (cholecystitis) was deemed related to ETI use, and two complications (obstetric cholestasis, *n* = 1 and pre-eclampsia in a 31-year-old woman with baseline ppFEV1 of 29%) were deemed of unknown relatedness to ETI use. Again, as was the case in women exposed to previously approved modulators during pregnancy, clinical decline was reported in 5 of 6 women who discontinued ETI because of its unknown risks to the fetus, prompting resumption of therapy. While clinicians deemed no infant complications as definitively related to ETI use, complications in 3 infants were deemed of unknown relatedness to ETI use: *n* = 1 infant who experienced transient transaminitis in addition to a choroid plexus cyst and uretocele, *n* = 1 infant born to a mother with CF-related diabetes (CFRD) who had low set ears, and *n* = 1 infant born to a mother with CFRD with mild aortic coarctation. Based on the mother’s past medical history, clinicians reported two severe congenital anomalies that they deemed unrelated to ETI use including 1 instance of Trisomy 16 in a mother with a history of two previous miscarriages prior to ETI use, and 1 instance of multiple malformations noted on prenatal ultrasound (resulting in pregnancy termination) in a woman with poorly controlled CFRD.

### 3.5. Considerations for Infants Exposed to CFTR Modulators during Pregnancy

For women who choose to continue modulators throughout pregnancy, adult providers should consider providing them with specific recommendations based on the infant’s exposure during pregnancy. Ideally, this information would be communicated to both the woman with CF and to her child’s pediatrician so that shared decision making could occur. First, although the partners of most women with CF undergo genetic testing for CF prior to pregnancy, not all may do so, and up to 50% of pregnancies in women with CF are reported to be unplanned [60]. Importantly, in the CF ferret animal model, when IVA was administered throughout pregnancy to ferrets with the IVA-responsive G551D (Gly551Asp) mutation, investigators demonstrated restoration of pancreatic function in kits [61]. Pancreatic sufficiency with a resultant false negative newborn screen in an infant homozygous for F508del exposed to ETI throughout pregnancy was recently reported in NY [62]. Because many other states also use a measure of pancreatic function to test for CF on newborn screening evaluation, maternal use of a highly effective modulator during pregnancy may also result in a false negative screen in an infant with CF. Therefore, if a father’s genotype is unknown, or if the father is known to be a CF carrier, the child should undergo genotyping following a negative newborn screening test. Second, in addition to counseling women with CF about potential false negative newborn screening tests, based on the data demonstrating cataract formation in juvenile rats exposed to IVA and the prescribing information guidance that infants and children taking ivacaftor undergo baseline and follow-up ophthalmologic exams [45], the mother should be advised to consider an ophthalmologic exam for her infant. The final issue about which providers caring for women with CF should counsel mothers (and communicate to the infant’s pediatrician) is the possible need for liver function testing in the infant. Although Trimble and colleagues reported high levels of LUM and IVA in cord blood in the mother who continued LUM/IVA throughout pregnancy, the infant’s liver function testing was normal at birth [49]. Thus, if the mother is not planning to breastfeed, and therefore will not continue to expose the infant through lactation, liver function testing may be unnecessary. However, if the mother does plan to continue her CFTR modulator therapy during breast feeding (see Section 4), a plan for standing or reflexive liver function testing in the infant could be considered.

In summary, based on the data from CFTR modulator administration in reproductive models and the limited data available in women with CF, the European Respiratory Society/Thoracic Society of Australia and New Zealand categorized CFTR modulators as “probably safe” during pregnancy [63]. However, to enable CF care providers to more definitively counsel women regarding the potential risks and benefits of use of CFTR modulators during pregnancy, a large, prospective study is needed.

## 4. Lactation

As women with CF are increasingly experiencing pregnancy, more questions are arising surrounding lactation, particularly in regards to medication safety. Historically, women and CF care providers were most concerned about energy expenditure of the mother and whether she would be able to maintain an appropriate nutritional status to stay healthy and avoid malnutrition and weight loss while breast feeding [64]. Lactation does require increased calorie intake and was often discouraged by CF care teams in the past. Weight loss postpartum can be rapid with some returning to their pre-pregnancy weight within the first 6 weeks postpartum [65]. With the advent of highly effective modulators, while people with CF are still pancreatic insufficient, their overall weight is improving and nutritional deficiency is much less common [6,10,11,66].

As with safety of medication use in pregnancy, reviews and recommendations for medications frequently used in lactating women with CF are published [50,51,63]. However, these recommendations are not guided by methodical studies and data collection. The most commonly used CF-related medications, such as pancreatic enzyme and airway clearance therapies, are generally considered safe to use during lactation, but extensive data are lacking. Data related to CFTR modulators and lactation remain extremely limited. In a case report of a woman who continued on her CFTR modulator during pregnancy and lactation, both IVA and LUM were shown to be excreted in breastmilk at subtherapeutic levels [49]. There were two transient elevations of bilirubin and liver enzymes in this breast fed infant, although the relationship to modulator exposure during breastfeeding was unclear. With the knowledge that CFTR modulators can cause elevated liver enzymes in people with CF taking these medications, infant monitoring of these measures during breastfeeding may be considered. In a survey in which data was collected from 64 pregnancies by Nash and colleagues on women with CF who continued IVA, LUM/IVA or TEZ/IVA, no modulator-related complications were reported in the twenty-seven infants exposed in utero and/or during lactation [56]. A recent study we conducted similarly found no adverse effects of ETI exposure during breastfeeding on 26 infants although the collected information was limited [35]. Overall, continuation of CFTR modulators during lactation will require a risk benefit discussion between the CF care team, OB team, infant pediatrician and the mother with CF, but as of current data, there is no clear harm to the infant if the mother continues to breast feed while taking CFTR modulator therapy.

## 5. Conclusions

We have entered an era in the care of people with CF in which the majority of people are eligible for highly effective CFTR modulators. Data from phase III clinical trials, case reports and case series suggest that use of CFTR modulators increases fertility in women with CF. Thus, clinical care providers must offer contraceptive counseling for young women who start CFTR modulators who wish to avoid pregnancy. On the other hand, with improved health status and expected longevity, more women with CF are expressing a desire to have children, and pregnancy rates are increasing. Pregnant and lactating women were excluded from Phase III trials of modulators. Thus, although data from animal models, case reports and case series has not shown alarming rates of miscarriage or other pregnancy or infant complications, prospective trials are needed to provide evidenced based recommendations to women with CF who are contemplating pregnancy. Until such data is available, clinicians and women with CF must continue to weigh the potential risk of clinical decline for the mother who chooses CFTR modulator discontinuation versus the potential unidentified risk to the developing fetus of continuing CFTR modulation. To assist the CF community with better data to guide use of CFTR modulators during pregnancy and lactation, beginning in late 2021, Drs. Jain and Taylor-Cousar will lead a multi-site prospective U.S. study, funded by the CF Foundation, to evaluate Maternal and FetaL Outcomes in the ERa of ModulatorS (MAYFLOWERS, NCT04828382).

## Figures and Tables

**Table 1:** Impact of CFTR modulator administration in animal reproductive models.

	Impaired Fertility	Genotoxicity	Teratogenicity	Neonatal Cataracts	Presence in Breast Milk
**Ivacaftor**	Yes at toxic human doses	None	At maternally toxic doses: ↓ fetal body weight; no impact on survival or organogenesis	Cataracts observed at all doses administered to juvenile rats	Yes *
**Lumacaftor**	No	None	No	When using combination therapy (i.e., LUM/IVA), see IVA	Yes *
**Tezacaftor**	No	None	At maternally toxic doses: ↓ fetal body weight, early development delay in pinna detachment/eye opening; no impact on survival or organogenesis	When using combination therapy (i.e., TEZ/IVA), see IVA	Yes
**Elexacaftor**	Yes at toxic human doses	None	At maternally toxic doses: ↓ fetal body weight; no impact on survival or organogenesis	When using combination therapy (i.e., ELX/TEZ/IVA), see IVA	Yes

Each modulator has been tested individually in animal reproductive models rather than in combination [45,46,47,48]. * Also observed in a case report of a human pregnancy during which LUM/IVA was continued during pregnancy and lactation [49]. ↓ = decreased.
